# RNAa-Mediated Gene Activation in the Regulation of Stem Cell Fate

**DOI:** 10.3390/biom16010005

**Published:** 2025-12-19

**Authors:** Hyohi Lee, Jiin Moon, Seung-Kyoon Kim

**Affiliations:** Department of Convergent Bioscience and Informatics, and Graduate School of Biological Sciences, Chungnam National University, Daejeon 34134, Republic of Korea

**Keywords:** RNA activation (RNAa), small activating RNA (saRNA), endogenous RNA activator, stem cell pluripotency, differentiation, epigenetic regulation

## Abstract

Stem cell fate is governed by complex transcriptional networks and dynamic chromatin architectures, with RNA molecules acting as critical regulators. Traditionally, small RNAs have been associated with gene silencing; however, growing evidence reveals that certain RNA species can also activate transcription, a phenomenon termed RNA activation (RNAa). This evolutionarily conserved mechanism functions through both synthetic small activating RNAs (saRNAs) and endogenous RNA molecules, including promoter-targeting microRNAs, small modulatory double-stranded RNAs, and circular RNAs. By modulating chromatin accessibility and engaging the transcriptional machinery, these RNAs orchestrate gene expression programs that control pluripotency maintenance and lineage-specific differentiation in stem cells. This review integrates emerging mechanistic insights and functional evidence to provide a comprehensive perspective on RNAa-mediated gene activation in stem cell biology and highlights its potential as a precise tool for controlling cell fate through epigenetic modulation.

## 1. Introduction

Small double-stranded RNAs have traditionally been studied for their gene-silencing functions through RNA interference (RNAi) [1,2,3]. The discovery of RNA activation (RNAa) as a functional counterpart to RNAi [4] challenged the conventional view of small RNAs as repressors [5,6] and established RNAa as an evolutionarily conserved transcriptional activation mechanism [7]. Since its discovery nearly two decades ago, RNAa has expanded the functional landscape of small RNAs by demonstrating that these molecules can act as transcriptional activators rather than merely repressors [4,7,8]. RNAa is an epigenetic process in which small RNA molecules, particularly small activating RNAs (saRNAs), interact with promoter regions to induce transcriptional activation of specific endogenous genes [8,9,10,11]. Mechanistically, RNAa operates through epigenetic modulation, including chromatin remodeling, histone modification, and RNA polymerase II (RNAPII) recruitment at target promoters, linking small RNA activity directly to transcriptional control. As such, RNAa has emerged as a powerful approach for understanding gene regulation and for modulating cell fate in therapeutic contexts [11,12,13,14].

Stem cells are characterized by their long-term self-renewal capacity and the ability to differentiate into multiple lineages, a property referred to as pluripotency [15,16]. Regulation of stem cell fate relies on complex molecular networks that integrate transcriptional control, chromatin dynamics, and epigenetic cues [17,18,19,20]. Extrinsic factors such as cell–cell interactions, the microenvironment, or external signaling pathways can reprogram these networks, leading to lineage-specific gene expression and differentiation [21,22,23]. While transcription factors and chromatin-modifying enzymes have traditionally been considered the central regulators of gene expression [24,25,26], RNA molecules are increasingly recognized as essential modulators of cell identity and developmental potential [27,28,29]. Stem cells provide an ideal model for studying RNAa, as their transcriptional states are highly plastic and sensitive to epigenetic modulation, allowing for precise examination of how small RNAs can reprogram gene expression and influence fate transitions.

Among these RNA-based mechanisms, RNAa provides a unique regulatory layer that enables targeted activation of endogenous genes without the need to introduce exogenous transcription factors or permanent genetic modifications [30,31]. Such precise control is particularly important in stem cell biology, where balanced gene expression determines whether cells maintain pluripotency or initiate lineage commitment. Recent studies have demonstrated that saRNAs can activate not only core pluripotency genes but also lineage-specific genes involved in differentiation [11,31]. These findings suggest that RNAa may serve as a versatile and safe tool for stem cell manipulation, with potential applications in regenerative medicine, disease modeling, and cell-based therapies [32,33,34].

In this review, we summarize the mechanistic principles of RNAa and its emerging roles in stem cell regulation. We focus on how both synthetic and endogenous small RNA systems contribute to transcriptional activation processes that govern the maintenance of pluripotency and the execution of lineage-specific differentiation.

## 2. Mechanisms of RNAa-Mediated Gene Activation

Small RNAs have traditionally been associated with gene silencing, particularly through RNAi pathways mediated by small interfering RNAs (siRNAs) and microRNAs (miRNAs) [35,36]. These molecules guide Argonaute (Ago) proteins to complementary mRNA targets, resulting in transcript degradation or translational repression [37,38]. However, a paradigm-shifting discovery in the early 2000s revealed that small double-stranded RNAs (dsRNAs), when designed to target promoter regions, can instead activate gene transcription. This unexpected phenomenon is known as RNAa [4].

RNAa was first described by Li et al. (2006), who demonstrated that promoter-targeting dsRNAs could induce transcriptional upregulation in human cells, thereby establishing RNAa as a sequence-specific mechanism for endogenous gene activation [4]. Subsequent studies have shown that RNAa is not restricted to synthetic saRNAs, but can also be mediated by endogenous ncRNAs, indicating that promoter-targeted small RNAs can act as positive regulators of transcriptional output [39]. Collectively, these findings support the view that RNAa is an evolutionarily conserved regulatory mechanism, observed across diverse eukaryotic systems including mammals, plants, and insects [4,7,40,41,42,43].

**Figure 1:**
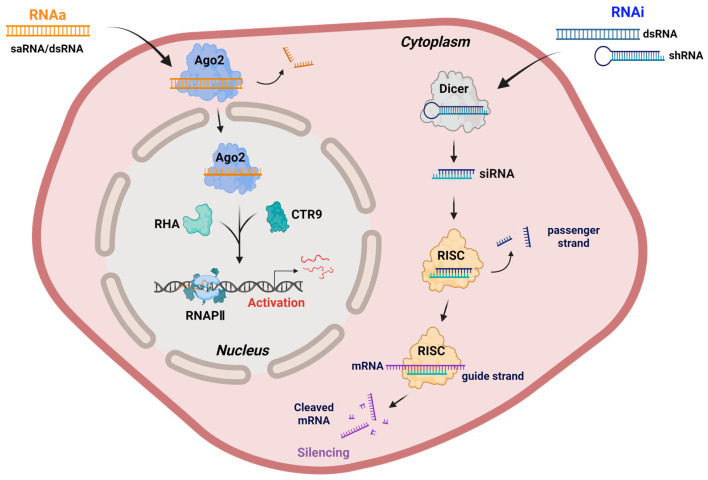
Mechanistic comparison of RNAa and RNAi. RNAa involves the binding of saRNAs or promoter-targeting dsRNAs to Ago2 in the nucleus, where it forms an activating complex with RHA and CTR9, leading to the recruitment of RNAPII and transcriptional activation of target genes. In contrast, RNAi is initiated by the processing of exogenous dsRNAs or shRNAs by Dicer into siRNAs, which are incorporated into the RISC in the cytoplasm, composed of core subunits such as Dicer, TRBP, and Ago2. The guide strand in RISC directs mRNA cleavage and degradation, leading to gene silencing. Although RNAi can contribute to heterochromatin formation and transcriptional repression in the nucleus under specific conditions, its primary site of action remains the cytoplasm. Note: Abbreviations: Ago2, Argonaute 2; CTR9, CTR9 component of the polymerase-associated factor complex; RHA, RNA helicase A; RNAPII, RNA polymerase II; RISC, RNA-induced silencing complex; siRNA, small interfering RNA. Created in Biorender. Lee, H. (2025) https://app.biorender.com/illustrations/68afd2ff58b546446ba9a294?slideId=d644e283-3965-4d0f-98bf-b27d89625255.

At the molecular level, RNAa shares some mechanistic components with RNAi but differs markedly in both outcome and subcellular localization (Figure 1). While RNAi primarily occurs in the cytoplasm to silence mRNAs, it can also contribute to heterochromatin formation in the nucleus under specific contexts [44,45,46]. In contrast, RNAa is initiated in the nucleus, where saRNAs or endogenous noncoding RNAs (ncRNAs) form a complex with Ago proteins, particularly Ago2 [8,9,47,48]. Recent studies suggest that Ago1 may also participate in promoter–proximal RNAa events in the nucleus, potentially complementing the Ago2-dependent pathway. This raises the possibility of functional specialization among different Ago family members in promoter-associated RNA regulation [49,50].

The resulting saRNA-Ago2 complex recognizes complementary promoter sequences or promoter-associated noncoding transcripts of target genes. This interaction promotes the recruitment of transcriptional co-activators, notably the RNA-induced transcriptional activation (RITA) complex, which comprises RNA helicase A (RHA) and CTR9, a subunit of the polymerase-associated factor 1 (PAF1) complex, a transcriptional co-regulator associated with RNAPII elongation. RHA facilitates chromatin remodeling and RNA-DNA hybrid resolution at the promoter, whereas CTR9 mediates downstream histone modifications associated with transcriptional activation [51,52]. In addition to histone H3 lysine 4 tri-methylation (H3K4me3) enrichment, RNAa has been associated with increased histone H3 acetylation and promoter DNA demethylation [53,54,55,56], further supporting its role in establishing an open chromatin conformation conducive to increased RNAPII occupancy and productive transcription [39,52,57]. Together, these interactions functionally bridge the saRNA-Ago2 complex with RNAPII, thereby enhancing transcriptional initiation and productive elongation [32,52,58].

Emerging evidence also points to a functional interplay between RNAa and promoter-associated long noncoding RNAs (lncRNAs). In several loci, lncRNAs or promoter-associated transcripts appear to serve as scaffolds that tether saRNA-Ago complexes to specific chromatin sites, reinforcing the transcriptional activation of neighboring genes [59,60]. This cooperative mechanism broadens the regulatory landscape of RNAa beyond direct promoter targeting, integrating it into larger RNA-based transcriptional networks.

Although only a subset of small RNAs participates in RNAa, mechanistic studies have identified several shared features of Ago-dependent, promoter-targeted activation that help explain this selectivity [58,61]. First, double-stranded small RNAs are processed in a manner similar to siRNAs: the duplex is unwound and the antisense guide strand is selectively loaded onto Ago proteins, most prominently Ago2. Guide-strand selection depends on sequence and thermodynamic properties, such as 5′-end stability and GC content, so only duplexes with favorable asymmetry and an appropriate seed sequence are efficiently incorporated into Ago and maintained in an active conformation [62,63]. Second, small-RNA loading stabilizes Ago2 and is often required for its nuclear accumulation. Ago and GW/TNRC6-family proteins have been shown to shuttle between the cytoplasm and nucleus, and Ago-small RNA-TNRC6 assemblies form a transport-competent unit for nuclear import, indicating that only Ago complexes that successfully acquire a guide strand and the necessary cofactors are competent for nuclear entry [64,65]. Third, once in the nucleus, Ago-small RNA complexes are recruited to specific loci through seed-region complementarity to promoter DNA or promoter-associated noncoding transcripts [60,66]. Taken together, these observations support a model in which small RNAs that are efficiently loaded onto Ago, enter the nucleus in association with appropriate Ago-associated cofactors, and exhibit high-fidelity seed complementarity to promoter-proximal targets are preferentially able to nucleate assembly of the RITA complex containing RHA, CTR9/PAF1, RNAPII, and associated histone-modifying enzymes, thereby driving RNAa at their target loci.

Beyond its mechanistic novelty, RNAa has been investigated in various biological and therapeutic contexts, including cancer, metabolic disorders, and tissue regeneration (Table 1) [67,68,69]. More recently, it has been applied to developmental and stem-cell-related genes, facilitating reprogramming and lineage-specific gene activation [31,68,70]. Collectively, RNAa represents a distinct transcriptional regulatory pathway that integrates sequence specificity with epigenetic modulation. A deeper understanding of its molecular basis will be essential for developing RNAa as a precise and programmable tool for gene control in both basic and translational research.

### 2.1. Ago-Dependent RNA Activators

#### 2.1.1. miRNAs

miRNAs are small ncRNAs approximately 21–23 nucleotides in length that primarily function as post-transcriptional repressors of gene expression by binding to complementary sequences in the 3′ untranslated regions (3′ UTRs) of target mRNAs [74]. For instance, members of the let-7 family target pluripotency-associated genes such as *Myc* and *Lin28*, and their upregulation promotes differentiation, thereby contributing to the loss of stem cell identity [75,76,77]. These interactions typically lead to translational inhibition or mRNA degradation mediated by Ago proteins within the RNA-induced silencing complex (RISC), which is composed of key subunits such as Dicer, TAR RNA-binding protein (TRBP), and Ago2 [74,78].

Although miRNAs are conventionally known for their silencing functions, accumulating evidence indicates that they can also activate gene transcription through RNAa mechanisms [6,79,80]. In this noncanonical role, miRNAs bind to complementary sequences within gene promoters or promoter-associated ncRNAs, thereby facilitating transcriptional upregulation [39,58,81]. A well-characterized example is miR-373, which activates transcription of E-cadherin and CSDC2 by targeting their promoter regions [39]. Nuclear localization of miRNAs and Ago proteins has been demonstrated as a prerequisite for this transcriptional activation. Specific subsets of Ago proteins, including Ago1 and Ago2, translocate into the nucleus to form miRNA-Ago complexes that recognize complementary promoter regions or nascent promoter-derived transcripts, thereby linking cytoplasmic RNA silencing machinery to nuclear transcriptional control [59,82].

Promoter-targeting miRNAs can induce RNAa through multiple mechanisms. In some cases, they recruit Ago proteins to promoter regions, leading to the enrichment of RNAPII and the accumulation of active histone modifications such as H3K4me3, which promote transcriptional initiation and gene activation [81]. Additional studies have shown that RNAa-associated promoter activation is also accompanied by a reduction in repressive histone marks, including loss of histone H3 lysine 9 di- and tri-methylation (H3K9me2/3), together with promoter DNA demethylation, collectively contributing to an open chromatin configuration that favors transcriptional engagement [4,54,61]. In other contexts, miR-34a facilitates the release of paused RNAPII through interactions with regulatory factors such as the DDX21–CDK9 complex, thereby enhancing transcriptional elongation [82]. Certain miRNAs can also directly engage core promoter elements, such as the TATA box, to promote transcription initiation [80]. Additionally, autoregulatory feedback loops have been described, exemplified by the lin-4 miRNA in C. elegans, which enhances its own transcription by interacting with its promoter region [6].

Beyond such self-regulatory circuits, several studies have demonstrated that a subset of miRNAs can localize to the nucleus and activate transcription through direct promoter targeting. Moreover, the identification of miR-140 as a key regulator of preadipocyte differentiation, acting through the lncRNA NEAT1 as a downstream effector, indicates that a miRNA-lncRNA regulatory axis can be functionally integrated into differentiation programs [33]. These observations together suggest that nuclear miRNA activities may contribute to the fine-tuning of lineage-specific gene induction as well as pluripotency-associated transcriptional networks. Taken together, these observations highlight the functional versatility of miRNAs, indicating that they act not only as canonical repressors in the cytoplasm but also as noncanonical activators of gene expression within the nucleus.

#### 2.1.2. saRNAs

saRNAs are synthetic double-stranded RNAs, typically 19–21 nucleotides in length, that target specific promoter regions to stimulate gene expression at the transcriptional level [4,83,84,85]. Li et al. (2006) first demonstrated this phenomenon by showing that saRNAs designed against the E-cadherin promoter, namely dsEcad-302 and dsEcad-215, significantly upregulated gene expression in human cells [4]. Unlike siRNAs, which are loaded symmetrically into the RISC, saRNAs display strand selectivity, with only the guide strand directing promoter recognition and nuclear Ago recruitment [4,8]. This antisense strand orientation is critical for target specificity and efficient transcriptional activation.

One of the best-characterized mechanistic models of RNAa involves studies on the human CDKN1A (p21) promoter [52]. In this system, saRNAs guide Ago2 to the promoter, where they recruit the RITA complex, which includes RHA and CTR9, a subunit of the PAF1 complex. This interaction is seed region-dependent. For instance, in the progesterone receptor (PR) model, mismatches in the 5′ seed region of the antisense strand or in the target promoter DNA markedly reduced RNAa activity, underscoring the importance of sequence complementarity between the antisense strand and the promoter DNA [86]. Upon nuclear entry, the saRNA-Ago2–RITA complex assembles at the target promoter, and this complex has been shown to enhance activating chromatin marks and RNAPII at the locus [4,8,52].

The RITA-Ago2 assembly associates with RNAPII to promote both transcriptional initiation and elongation, accompanied by histone H2B lysine 120 ubiquitination (H2BK120ub) and downstream histone H3 lysine 4 and 79 trimethylation (H3K4me3 and H3K79me3), which serve as epigenetic marks of active transcription [52,87,88]. Additionally, saRNAs enhance the recruitment of initiating RNAPII near the transcription start site, followed by increased Ser2 phosphorylation across the gene body, which indicates a transition from polymerase pausing to productive elongation mediated through Ago2 interaction [52].

Beyond RITA-mediated activation, saRNAs also influence transcription through additional chromatin-based and RNA-guided mechanisms [84]. In human fibroblasts, saRNAs have been shown to induce nucleosome repositioning at target promoters, thereby increasing promoter accessibility for transcription factors [57]. Moreover, saRNA activity has been functionally linked to the activation of lineage- and pluripotency-related genes, demonstrating potential applications in stem cell reprogramming and regenerative medicine, where transient and locus-specific activation of endogenous genes is desirable [11,68,89].

In parallel with these mechanistic and functional studies, several practical design principles and computational workflows for saRNAs have begun to emerge. Most active saRNAs reported to date have been identified empirically, but early work recommended designing panels of duplexes that tile a region upstream of the transcription start site (TSS), typically between approximately −100 and −1000 bp, while avoiding CpG islands and repetitive elements, maintaining moderate GC content, and preserving an antisense guide strand with favorable thermodynamic asymmetry for Ago loading [48,85]. Analyses of larger sets of activating and non-activating duplexes further highlighted the importance of seed-region complementarity to the promoter and local chromatin features at the target locus in determining RNAa potency [48,66,85]. Dedicated resources are also beginning to appear: saRNAdb, for example, is a curated database of experimentally validated saRNAs that stores their sequences, promoter binding locations, predicted features, and associated proteins, providing a useful starting point for motif discovery and training of design algorithms [83]. In addition, small-RNA off-target analysis frameworks such as SeedMatchR1.1.1, an R package that quantifies seed-mediated transcriptional effects and can detect potential activating seed matches when promoter sequences are supplied as targets, illustrate how transcriptome-scale computational tools can be repurposed to study RNAa [90]. Together, these developments show that computational approaches can narrow the search space for candidate saRNAs and help manage off-target risk, although design frameworks capable of consistently predicting strongly activating saRNAs are still not fully mature.

In parallel with these computational advances, progress in chemical synthesis and delivery technologies has supported the experimental and therapeutic use of synthetic saRNAs. To improve stability in biological environments and reduce innate immune activation, chemical modifications originally developed for siRNA platforms have also been applied to saRNAs. Common strategies include 2′-O-methyl (2′-OMe) and 2′-fluoro (2′-F) substitutions at selected positions, which enhance nuclease resistance, reduce pattern-recognition receptor binding, and increase duplex stability [91,92].

For in vitro studies, saRNAs are typically delivered using cationic lipids or liposome-based transfection reagents to introduce promoter-targeting RNAs into cells, thereby inducing RNAa through endogenous Ago2-mediated mechanisms [4,93]. In preclinical and translational settings, lipid-based nanoparticles (LNPs) and other nanocarriers have been used to deliver saRNA molecules, with several studies reporting target gene activation or therapeutic benefits following systemic administration. For instance, LNP formulations similar to those used in FDA-approved siRNA therapeutics have been optimized for targeted delivery of saRNAs in vivo [72,94]. One notable example involves a first-in-human clinical candidate in which CEBPA-saRNA was encapsulated in LNPs to restore CEBPA expression and suppress tumor progression in hepatocellular carcinoma models [94]. More recently, a transferrin receptor-targeting DNA nanostructure was employed to deliver CEBPA-saRNA specifically to pancreatic cancer cells, resulting in significant CEBPA activation and antitumor effects in vivo [95]. In addition to these approaches, delivery platforms such as polymeric nanoparticles, cell-penetrating peptides, and exosome-based carriers have been explored in related RNA delivery contexts and may also be applicable to saRNA-mediated activation, particularly for achieving cell type-specific targeting while minimizing off-target immune responses [96,97]. Importantly, tissue or cellular distribution of saRNAs is not an inherent property of the RNA molecules themselves, but is instead determined by the delivery platform and route of administration. To date, most preclinical and early-phase clinical studies have focused on liver and tumor models using LNP-based delivery of CEBPA-saRNA. In other cell types, including immune cells and stem cells, delivery efficiency is more variable and less well characterized. Therefore, defining the most efficient saRNA carrier for specific cell types remains an open question and a key direction for future research. In parallel, studies of CEBPA-saRNA in liver and pancreatic cancer indicate that saRNA-mediated activation of tumor-suppressor genes can suppress CSC-like phenotypes, while careful sequence design and off-target evaluation are essential to avoid unintended activation of oncogenic promoters.

Collectively, these mechanistic, functional, and computational advances demonstrate that saRNAs act as potent regulators of transcription, not by silencing genes like conventional small RNAs, but by reprogramming chromatin structure and transcriptional dynamics to activate endogenous gene expression. Alongside these discoveries, the development of chemically stabilized saRNA duplexes and lipid-based delivery platforms has enabled their use in both mechanistic studies and emerging therapeutic applications, further supporting the translational potential of RNAa.

### 2.2. Other RNA Activators

In addition to miRNA- and saRNA-guided RNAa, several classes of promoter-associated ncRNAs, including smRNAs, circRNAs, and pancRNAs, have also been reported to induce RNAa-like transcriptional activation, in part through interactions with chromatin-associated regulators.

#### 2.2.1. Small Modulatory RNAs (smRNAs)

smRNAs are endogenous double-stranded noncoding RNAs of approximately 20 base pairs in length that regulate gene expression by targeting promoter sequences [70,85]. Unlike siRNAs or miRNAs that predominantly function in the cytoplasm, smRNAs are typically derived from promoter-associated or natural antisense transcripts and exert their effects within the nucleus, highlighting their unique role in transcriptional modulation [8,70]. A representative example is the neuron-restrictive silencer element (NRSE, also known as RE1)-derived smRNA identified in adult neural stem cells, which binds complementarily to the NRSE sequence and interacts with the RE1-silencing transcription factor (REST) complex. This interaction facilitates the displacement of transcriptional repressors such as histone deacetylase (HDAC) and MeCP2 from the promoter region while recruiting co-activators such as CBP/p300. The process is accompanied by increased histone acetylation at the target locus, resulting in a transition from a repressive to a transcriptionally permissive chromatin state [70]. Functionally, activation of REST-regulated neuronal genes by NRSE-derived smRNAs promotes neural differentiation, underscoring the role of smRNA-mediated RNAa in lineage-specific transcriptional reprogramming. These findings illustrate how small double-stranded RNAs can modulate gene activation through coordinated RNA–protein–DNA interactions and local chromatin remodeling.

#### 2.2.2. Circular RNAs (circRNAs)

circRNAs constitute a unique and evolutionarily conserved class of endogenous noncoding transcripts characterized by a covalently closed loop structure lacking 5′ caps and 3′ poly(A) tail [98]. They are generated through a back-splicing mechanism in which a downstream splice donor is joined to an upstream splice acceptor, producing a closed circular transcript that exhibits remarkable stability due to exonuclease resistance [99,100,101]. One of their best-known functions is acting as miRNA sponges in the cytoplasm, where they sequester miRNAs and indirectly regulate gene expression [102,103]. A well-studied example is Cdr1as (also known as CiRS-7), which contains multiple binding sites for miR-7 and effectively sequesters it. This sequestration relieves the repressive effect of miR-7 on its target mRNAs, leading to indirect upregulation of gene expression, a mechanism implicated in several disease models including myocardial infarction [104].

More recently, circRNAs have also been shown to directly activate nuclear gene transcription through mechanisms reminiscent of RNAa. In this context, circRNAs act as scaffolds that bridge RNA–protein–DNA interactions at promoter regions, paralleling the saRNA-Ago2-RITA complex seen in classical RNAa pathways [105,106]. One such mechanism involves exon–intron circRNAs (EIciRNAs), a subclass that retains intronic sequences and localizes to the nucleus. These EIciRNAs, such as circEIF3J and circPAIP2, bind to the promoter regions of their host genes and physically associate with both RNAPII and U1 snRNP, thereby stabilizing the transcriptional complex at the promoter and enhancing *cis*-acting transcriptional activation [105]. Another subclass, circular intronic RNAs (ciRNAs), also localizes to the nucleus and regulates transcription in *cis*. For example, ci-ankrd52 accumulates at its transcription site and interacts with elongating RNAPII to promote transcriptional elongation. Knockdown of ci-ankrd52 reduces the expression of its host gene, indicating its role as a positive regulator of RNAPII activity [107].

Notably, several circRNAs have been implicated in stem cell regulation. For instance, circBIRC6 maintains pluripotency in human embryonic stem cells by sponging miR-34a and miR-145, whereas circFOXP1 preserves mesenchymal stem cell identity by inhibiting microRNAs involved in differentiation. Together, these findings raise the possibility that circRNA-based regulatory modules may extend to RNAa-like promoter control mechanisms in stem cell self-renewal and lineage specification [108,109]. Collectively, the covalently closed structure of circRNAs supports their function as stable and versatile regulators of gene expression, enabling fine-tuned transcriptional activation through modulation of both initiation and elongation processes.

#### 2.2.3. Promoter-Associated Noncoding RNAs (pancRNAs)

Promoter-associated ncRNAs (pancRNAs, also known as upstream transcripts (PROMPTs) are a class of noncoding transcripts ex-pressed in close proximity to TSS [110,111,112]. They are typically transcribed bidirectionally from active promoter regions, similar to enhancer RNAs (eRNAs) [113,114,115,116], often overlapping with promoter-associated antisense transcripts, and are thought to arise as byproducts of RNAPII initiation events [117,118]. Unlike other long ncRNAs, pancRNAs exert predominantly *cis*-regulatory functions by modulating the transcriptional activity of adjacent genes [119].

Recent evidence suggests that pancRNAs promote gene activation by enhancing chromatin accessibility through epigenetic mechanisms. Specifically, pancRNA expression correlates with the enrichment of active histone marks such as H3K4 trimethylation and H3K27 acetylation at their corresponding promoters [111]. In rat cell lines, overexpression of single-stranded pancRNAs has been shown to induce sequence-specific DNA demethylation at target promoters, supporting a model in which pancRNAs remodel the local epigenetic landscape to favor transcriptional activation [120]. A representative mechanistic example involves the promoter-associated antisense RNA, paRCDH1-AS, transcribed from the Cadherin-1 (CDH1) locus. paRCDH1-AS acts as an RNA scaffold that sequesters epigenetic UHRF1, DNMT3A, SUV39H1, and SUZ12, thereby preventing their binding to the CDH1 promoter [121]. This mechanistic parallel with RNAa pathways supports the notion that pancRNAs can serve as endogenous mediators of promoter-directed, RNA-dependent gene activation.

Functionally, in developmental and stem cell contexts, pancRNAs have been proposed to modulate chromatin plasticity and lineage-specific gene activation, positioning them as crucial mediators that link promoter transcriptional activity with epigenetic reprogramming [117,122]. These observations position pancRNAs as important regulators that integrate transcriptional and epigenetic control at promoter regions to fine-tune gene activation.

**Figure 2:**
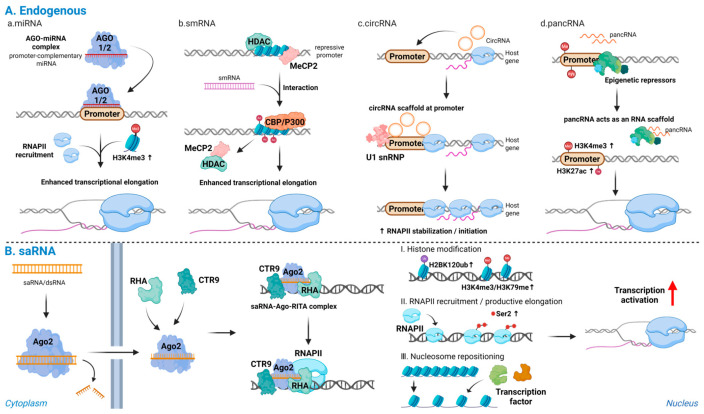
Endogenous and synthetic promoter-directed RNAs activate transcription: (**A**) Endogenous promoter-directed RNAs: (**a**) miRNAs; (**b**) smRNAs; (**c**) circRNAs; and (**d**) pancRNAs. These RNAs recognize promoter DNA or promoter-associated transcripts, displace repressive complexes, recruit transcriptional co-activators, and scaffold RNAPII or U1 snRNP at the target locus, leading to accumulation of active histone marks and stabilization of transcriptionally engaged RNAPII. (**B**) Synthetic saRNAs: promoter-complementary saRNAs are loaded onto Ago2 and, together with RHA and CTR9, form an activating complex at the target promoter, inducing H2BK120ub, followed by H3K4me3 and H3K79me3, enhanced RNAPII recruitment and Ser2 phosphorylation, nucleosome repositioning, and productive transcriptional activation of endogenous genes. Note: Abbreviations: Ago2, Argonaute 2; saRNA, small activating RNA; smRNA, small modulatory RNA; circRNA, circular RNA; pancRNA, promoter-associated noncoding RNA; RHA, RNA helicase A; CTR9, CTR9 subunit of the polymerase-associated factor (PAF1) complex; RNAPII, RNA polymerase II; H2BK120ub, monoubiquitination of histone H2B at lysine 120; H3K4me3, trimethylation of histone H3 at lysine 4; H3K79me3, trimethylation of histone H3 at lysine 79; RISC, RNA-induced silencing complex; siRNA, small interfering. Created in Biorender. Lee, H. (2025) https://app.biorender.com/illustrations/68afd2ff58b546446ba9a294?slideId=d644e283-3965-4d0f-98bf-b27d89625255.

Taken together, promoter-targeting RNAs of distinct origins converge on a common sequence of event-promoter recognition, local chromatin activation, and promotion of transcription initiation and elongation (Figure 2).

## 3. RNAa in Pluripotency Regulation

Pluripotent stem cells are defined by their dual capacities for self-renewal and potency, that is, the ability to proliferate indefinitely while retaining the potential to differentiate into all cell types derived from the three embryonic germ layers [123,124]. Pluripotency encompasses a spectrum of cellular states with distinct transcriptomic and epigenetic profiles that reflect early embryonic development and reprogramming processes [125,126]. The ectopic overexpression of key pluripotency transcription factors through viral vectors or modified mRNA has long been the primary strategy for reprogramming cell fate [127,128,129]. In contrast, RNAa-based approaches have emerged as an alternative strategy that enables locus-specific activation of endogenous gene expression without introducing exogenous genetic constructs, thereby providing a potentially safer and more targeted means of inducing cell fate transitions. To date, most RNAa applications in stem cell systems have employed synthetic saRNAs, typically 19–21-nucleotide double-stranded RNAs designed to target defined windows within the proximal promoter region of pluripotency-associated genes.

Core transcription factors such as OCT4, SOX2, KLF4, and c-MYC are essential regulators of pluripotency and are widely recognized as hallmarks of stem cell identity [128]. These factors orchestrate transcriptional networks that maintain self-renewal while repressing differentiation pathways. Because of their pivotal roles, the expression of these master regulators is tightly controlled; both overexpression and depletion can disrupt the delicate balance between maintaining pluripotency and initiating lineage commitment [128,130,131,132].

Within the context of RNAa-mediated regulation, a systematic screen of double-stranded RNAs designed to target the OCT4 promoter in human mesenchymal stem cells (MSCs) [31]. They designed a panel of 19-bp dsRNAs that tiled the ~1 kb region upstream of the human OCT4 transcription start site and identified a duplex designated dsOCT4-622, whose antisense strand targets position -622, as the most potent activator of OCT4 [31]. Functionally, dsOCT4-622 increased endogenous OCT4 mRNA expression in MSC types, and treatment with the histone deacetylase inhibitor valproic acid (VPA) further augmented OCT4 induction by the promoter-targeting saRNA, indicating that chromatin-relaxing conditions can synergize with RNAa-mediated activation. Moreover, co-delivery of the OCT4 saRNA together with viral vectors expressing SOX2, c-MYC, and KLF4 enabled MSCs to generate partially reprogrammed induced pluripotent stem cells (iPSCs) [31], indicating that a single well-validated saRNA can support pluripotency networks in primary stem cells in a manner consistent with the Ago2- and chromatin-based mechanisms.

**Figure 3:**
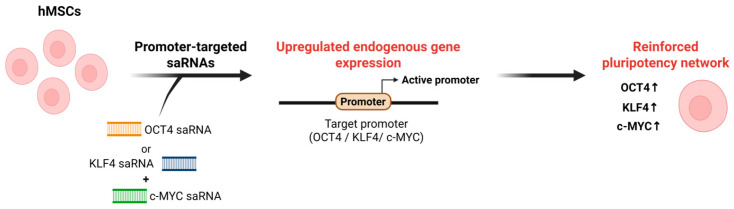
Schematic of RNAa-based activation of pluripotency regulators. In hMSCs, promoter-targeted saRNAs designed against the OCT4, KLF4, or c-MYC promoter are delivered into cells and recognize complementary sequences at the target promoter. This RNAa induces promoter activation and upregulation of endogenous OCT4, KLF4, and c-MYC expression, thereby reinforcing pluripotency-associated transcriptional networks in hMSCs. Note: Abbreviations: hMSC, Human mesenchymal stem cell. Created in Biorender. Lee, H. (2025) https://app.biorender.com/illustrations/68afd2ff58b546446ba9a294?slideId=d644e283-3965-4d0f-98bf-b27d89625255.

Similarly, Voutila et al. (2012) identified promoter-targeted saRNAs capable of activating *Klf4* and *c-Myc* transcription in human MSCs [133]. They designed panels of approximately 19-nt saRNA duplexes targeting the KLF4 and c-MYC promoter regions and, through iterative screening, identified KLF4-PR1 and MYC-PR1/MYC-PR2 as lead candidates that most effectively enhanced target gene expression. Transfection with these saRNAs induced robust, time- and dose-dependent upregulation of Klf4 and c-Myc at both the mRNA and protein levels compared with scrambled controls. Global transcriptome analyses revealed that saRNA-induced activation produced gene expression profiles resembling those observed with ectopic overexpression, particularly within stemness- and cell cycle-related pathways, underscoring the functional relevance of RNAa in maintaining pluripotency [133]. Importantly, these studies show that promoter-targeted saRNAs can upregulate core pluripotency genes in stem cells, providing functional evidence that RNAa can reinforce stem cell identity [31,133] (Figure 3). Together with reports showing that endogenous small RNAs can activate gene expression through promoter recognition [39,70], this implies that endogenous small RNAs may also contribute to RNAa-like transcriptional activation mechanisms underlying pluripotency and lineage specification.

Collectively, RNAa provides a precise and reversible means to regulate pluripotency-associated transcriptional networks (Table 2). By directly targeting promoter regions of endogenous master regulators, RNAa can fine-tune the balance between self-renewal and differentiation, offering a potential platform for safer, transgene-free reprogramming strategies in regenerative medicine and stem cell engineering.

**Table 2:** RNAa-mediated activation of pluripotency and lineage genes.

Context	System	Target	Result	References
hMSCs	Pluripotency	OCT4	OCT4-targeting saRNA increases OCT4 expression; VPA enhances this effect and supports partial iPSC reprogramming with SOX2, c-MYC, and KLF4.	[31]
hMSCs	Pluripotency	KLF4	KLF4/c-MYC saRNAs upregulate both genes in a dose- and time-dependent manner, producing a stemness- and cell cycle-biased profile.	
		c-MYC		[133]
ESCs	Cardiogenic differentiation	CDK9	CDK9-targeting small RNA upregulates CDK9 in an Ago/antisense-dependent way, increasing beating colonies and cardiac markers to promote cardiac differentiation.	[134]
Adipose-derived stem cells	Myogenic differentiation	MYOD	MYOD-targeting saRNA activates MYOD, elevates muscle markers, reduces proliferation, and enhances myogenic differentiation.	[135]
Neural stem cells	Neuronal differentiation	NRSE/RE1	NRSE dsRNA interacts with the REST complex to switch neuronal genes from repressed to active states and drives neuronal lineage commitment.	[70]

This table summarizes studies in which RNAa was used to activate endogenous regulators of stem cell state. In pluripotent or mesenchymal stem cells, saRNAs targeting OCT4 or KLF4/c-MYC increased the expression of core pluripotency and proliferation programs, particularly under chromatin-relaxing conditions. In differentiation contexts, cardiac, myogenic, and neuronal targets were activated at their native loci, leading to enhanced lineage marker expression and functional differentiation. Collectively, these examples demonstrate that RNAa can be applied not only to maintain or enhance pluripotency but also to drive directed differentiation through locus-specific activation of endogenous genes.

## 4. RNAa in Stem Cell Differentiation

Differentiation from a pluripotent state into specialized cell types involves a coordinated cascade of transcriptional, epigenetic, and signaling events [136,137,138]. In vitro, pluripotent stem cells can differentiate into derivatives of all three germ layers through the activation of lineage-specific gene expression programs [138,139], which are reinforced by distinct transcription factor networks [138,140]. Differentiation is typically initiated by signaling pathway outputs, including metabolic cues and growth factors, that promote the expression of lineage-determining genes [140,141]. Given the need for precise control of gene expression during this process, tools capable of selectively activating or repressing endogenous genes with high specificity hold considerable potential. In this context, RNAa-based strategies provide a means of inducing lineage-specific gene expression at endogenous loci, thereby enabling directed differentiation without the introduction of exogenous transgenes.

In a study on small RNA-directed epigenetic programming of cardiac differentiation in embryonic stem cells, a short noncoding RNA homologous to the Cdk9 transcript was found to promote lineage commitment toward a cardiac fate. Transfection with a 22-nucleotide small RNA derived from Cdk9 exon sequences significantly increased Cdk9 transcription and protein expression through an RNAa-like mechanism dependent on Ago proteins and an endogenous antisense transcript. Functionally, elevated Cdk9 expression enhanced cardiomyocyte differentiation, as evidenced by an increased number of beating colonies and the upregulation of cardiac-specific marker genes [134]. Similarly, activation of MyoD by promoter-targeted saRNAs in rat adipose-derived stem cells enhanced myogenic differentiation, characterized by elevated expression of muscle-specific markers such as desmin and reduced cell proliferation [135]. These examples collectively illustrate that RNAa can selectively activate lineage-determining transcriptional programs, linking promoter-targeted RNA activity to cell fate specification.

In addition to exogenously introduced saRNAs, endogenous small RNAs have also been shown to drive lineage-specific differentiation via transcriptional activation. A small noncoding double-stranded RNA corresponding to NRSE/RE1 was reported to trigger neuronal differentiation by converting transcriptional repression into activation [70]. This NRSE dsRNA interacts with the REST complex to shift neuronal genes from a silenced state in neural stem cells to an active state in early neurons. Notably, NRSE dsRNA was both necessary and sufficient to direct multipotent neural stem cells toward a neuronal lineage, demonstrating that endogenous dsRNAs can serve as intrinsic regulators of differentiation through transcriptional activation mechanisms distinct from canonical miRNA or siRNA pathways [70]. It is plausible that similar endogenous RNAa-like mechanisms operate broadly across developmental systems, where pancRNAs, which include both sense and antisense promoter transcripts, cooperate with Ago complexes to modulate lineage gene activation.

Altogether, these findings underscore RNAa as a versatile mechanism capable of reprogramming chromatin and transcriptional states to promote lineage commitment (Table 2). By enabling locus-specific activation of differentiation genes, RNAa-based approaches hold promise for controlled stem cell differentiation, tissue regeneration, and regenerative medicine applications.

## 5. Conclusions

RNAa has broadened the paradigm of RNA-based gene regulation by demonstrating that small RNAs can mediate transcriptional activation in addition to repression. Through the actions of synthetic saRNAs and certain endogenous RNA species, RNAa provides a mechanism for sequence-specific promoter targeting that stimulates gene expression via chromatin remodeling and recruitment of transcriptional machinery, without the need for DNA integration or transgene expression. This mechanism exemplifies a unique layer of epigenetic control, integrating RNA–protein–DNA interactions to fine-tune transcriptional output in a programmable manner.

In stem cell systems, RNAa has shown potential for both maintaining pluripotency and directing lineage-specific differentiation. For example, saRNAs targeting *OCT4*, *KLF4* and *c-MYC* effectively reactivate these core pluripotency regulators in human stem cells. During differentiation, RNAa-mediated activation of Cdk9 in embryonic stem cells enhances cardiac lineage commitment, while saRNAs targeting MyoD in adipose-derived stem cells promote myogenic differentiation. Moreover, endogenous double-stranded RNAs, such as the NRSE RNA, have been shown to activate neuronal gene expression in neural stem cells, suggesting that intrinsic RNA-guided transcriptional activation mechanisms may already operate within cells. Together, these findings underscore the versatility of RNAa as both an experimental tool and a physiological mechanism for reprogramming gene expression and cell fate.

Despite these advances, the overall progress of RNAa has been more modest than initially expected. Many of the mechanistic and stem cell focused studies highlighted in this review were published between 2006 and the early 2010s, and relatively few new in vivo or translational reports have appeared in the last five years. This slow pace likely reflects several practical bottlenecks that remain to be overcome, including incomplete rules for designing potent saRNAs, promoter and cell type dependent variability in responsiveness, modest and context dependent activation levels at some loci, and the lack of standardized, tissue specific delivery platforms for saRNAs. Off-target effects at the chromatin and transcriptional levels are also less systematically characterized than for other gene regulatory technologies, which may further limit broader adoption of RNAa.

In parallel, CRISPR activation (CRISPRa) has emerged as a widely adopted platform for programmable gene activation. RNAa relies on chemically synthesized or endogenously processed small RNAs that recruit Ago-containing complexes to promoters, and therefore offers very small, non-integrating cargos that can be delivered using siRNA like formulations without the need for expression of large effector cassettes. By contrast, CRISPRa uses nuclease dead Cas proteins fused to strong transcriptional activators that are guided by single guide RNAs and typically achieves higher and more robust levels of gene activation with greater computational predictability of guide design. However, CRISPRa requires delivery of large dCas activator constructs in addition to guide RNAs, which complicates in vivo delivery and may raise concerns about long-term expression and immunogenicity. Taken together, RNAa and CRISPRa should be viewed as complementary rather than competing approaches, with RNAa providing a compact, transient, transgene-free strategy and CRISPRa offering more potent but technically more demanding activation.

Building on these limitations and opportunities, future research should aim to delineate the genome-wide determinants of RNAa responsiveness, develop saRNA-specific computational design algorithms that incorporate promoter sequence and chromatin context, and establish in vivo delivery systems that can achieve tissue-specific activation with minimal off-target effects. Integration of RNAa with other programmable platforms, such as CRISPRa or epigenome editing technologies, may further expand its applicability and allow more flexible control of transcriptional programs. If these challenges are addressed, RNAa could become a foundational platform for precise modulation of gene expression in stem cell engineering, regenerative medicine, and disease modeling, enabling the design of cell fate control strategies with high specificity and minimal genomic perturbation.

## Figures and Tables

**Table 1:** Therapeutic and biological applications of RNAa.

Disease	Target	Results	References
Metabolic syndrome	SIRT1	Sirtuin 1 (SIRT1)-targeting saRNA activates SIRT1 expression, reduces inflammatory-like responses, and restores normal lipid metabolism.	[67]
Renal cell carcinoma	p21	p21-targeting dsRNA (dsP21) activates the p21 promoter, increases p21 expression, and induces G1-phase cell cycle arrest.	[71]
Loss of inner ear hair cells	ATOH1	ATOH1-targeting saRNA activates ATOH1 and promotes MSC differentiation into hair cell–like cells for regenerative purposes.	[68]
Cirrhotic liver hepatocellular carcinoma	CEBPA	CEBPA-targeting saRNA improves liver function and survival, and significantly reduces tumor burden in rodent models of cirrhosis-associated hepatocellular carcinoma.	[72]
Breast and ovarian cancers	p27	miR-124 activates the p27 promoter, elevates p27 protein levels, and induces G1-phase cell cycle arrest.	[73]

Representative studies targeting endogenous genes with promoter-directed small RNAs are summarized according to their biological or disease context, target gene, and observed outcome. RNAa has been applied in metabolic syndrome (SIRT1 saRNA) to reduce inflammatory responses and restore lipid metabolism; in renal cell carcinoma (dsP21) to induce p21 expression and promote G1-phase arrest; in inner ear hair-cell regeneration (ATOH1 saRNA) to drive differentiation into hair cell–like cells; in cirrhotic liver hepato-cellular carcinoma (CEBPA-targeting saRNA) to activate CEBPA and its downstream effector P21 and suppress tumor cell proliferation; and in breast and ovarian cancers (miR-124–mediated p27 activation) to enhance cell cycle blockade. Collectively, these examples demonstrate that RNAa can be utilized not only as a tool to probe gene function but also as a potential therapeutic approach for metabolic, regenerative, and anticancer applications. Note: Abbreviations: saRNA, Small activating RNA.

## Data Availability

No new data were created or analyzed in this study.

## Abbreviations

The following abbreviations are used in this manuscript:

RNAi	RNA interference
RNAa	RNA activation
saRNA	Small activating RNA
RNAPII	RNA polymerase II
Ago	Argonaute
siRNA	Small interfering RNA
miRNA	MicroRNA
dsRNA	Small double-stranded RNA
mRNA	Messenger RNA
TRBP	TAR RNA-binding protein
ncRNA	Noncoding RNA
lncRNA	Long noncoding RNA
HDAC	Histone deacetylase
RITA	RNA-induced transcriptional activation
RHA	RNA Helicase A
PAF1	Polymerase-associated factor 1
3′ UTR	3′ untranslated regions
TSS	Transcription start site
2′-OMe	2′-O-methyl
2′-F	2′-fluoro
LNP	Lipid-based nanoparticles
RISC	RNA-induced silencing complex
smRNA	Small modulatory RNA
NRSE	Neuron-restrictive silencer element
REST	RE1-silencing transcription factor
CircRNA	Circular RNA
EIciRNA	Exon–intron circRNA
pancRNA	Promoter-associated ncRNA
eRNA	Enhancer RNA
CDH-1	Cadherin-1
PR	Progesterone receptor
MSC	Mesenchymal stem cell
VPA	Valproic acid
iPSC	Induced pluripotent stem cell
CRISPRa	CRISPR activation
